# Baculovirus PTP2 Functions as a Pro-Apoptotic Protein

**DOI:** 10.3390/v10040181

**Published:** 2018-04-07

**Authors:** Yue Han, Stineke van Houte, Monique M. van Oers, Vera I.D. Ros

**Affiliations:** 1 Laboratory of Virology, Wageningen University & Research, Droevendaalsesteeg 1, 6708 PB Wageningen, The Netherlands; yue.han@wur.nl (Y.H.); monique.vanoers@wur.nl (M.M.v.O.); 2 Centre for Ecology and Conservation, Biosciences, University of Exeter, Penryn, Cornwall TR10 9FE, UK; vanhoute.stineke@gmail.com

**Keywords:** baculovirus, apoptosis, *Spodoptera exigua*, SeMNPV, protein tyrosine phosphatase 2, pro-apoptotic effects, caspase assay, hemocytes, Lepidoptera

## Abstract

The family *Baculoviridae* encompasses a large number of invertebrate viruses, mainly infecting caterpillars of the order Lepidoptera. The baculovirus Spodoptera exigua multiple nucleopolyhedrovirus (SeMNPV) induces physiological and behavioral changes in its host *Spodoptera exigua*, as well as immunological responses, which may affect virus transmission. Here we show that the SeMNPV-encoded protein tyrosine phosphatase 2 (PTP2) induces mild apoptosis in *Spodoptera frugiperda* (Sf) 21 cells upon transient expression. Transient expression of a catalytic-site mutant of *ptp2* did not lead to apoptosis, indicating that the phosphatase activity of PTP2 is needed to induce apoptosis. We also found that the caspase level (indicator of apoptosis) was higher in cells transfected with the *ptp2* gene than in cells transfected with the catalytic mutant. Adding a caspase inhibitor reduced the level of *ptp2*-induced apoptosis. Moreover, deletion of the *ptp2* gene from the viral genome prevented the induction of apoptosis in *S. exigua* hemocytes. The virus titer and virulence indices (the viral infectivity and the time to death) were not affected by deletion of the *ptp2* gene. However, the viral occlusion body yield from *S. exigua* larvae infected with the mutant virus lacking the *ptp2* gene was much lower than the yield from larvae infected with the wild-type (WT) virus. We hypothesize that the observed pro-apoptotic effects of PTP2 are the result of PTP2-mediated immune suppression in larvae, which consequently leads to higher viral occlusion body yields.

## 1. Introduction

Apoptosis is an active process of programmed cell death that is involved in immunity, normal development and cell differentiation [1]. It is characterized by morphological changes, including cell blebbing and cell shrinkage, chromosomal DNA fragmentation, as well as energy-dependent biochemical changes [1,2]. Apoptosis is part of the insect’s innate immune response against pathogens, including viruses, triggering premature cell death of infected host cells [3]. Viruses have developed a range of strategies to counteract host-induced apoptosis [3], including the expression of anti-apoptotic genes. On the other hand, some viruses have been found to actively induce apoptosis in their host in order to suppress the host immune system and/or enhance virus dissemination [4,5]. For example, it was found that the polydnavirus Microplitis demolitor bracovirus (MdBV) induced apoptosis in immune cells of *Spodoptera frugiperda* caterpillars and that this activity contributed to the immunosuppression of the host [5]. Several viral genes and proteins have been found to induce pro-apoptotic effects in host-derived cell lines [4,5,6,7,8]. For example, Suderman et al. [5] found that the protein tyrosine phosphatase-H2 (PTP-H2) from MdBV induced apoptosis in *S. frugiperda* 21 (Sf21) cells and showed that the phosphatase activity of PTP-H2 was needed for the apoptotic activity. The iridovirus serine/threonine kinase (ISTK) from Chilo iridescent virus (CIV) induced apoptosis in cell lines derived from the spruce budworm *Choristoneura fumiferana* and the boll weevil *Anthonomus grandis* [4].

Baculoviruses are arthropod-specific viruses with a circular, double-stranded DNA genome [9]. Two types of baculovirus virions are present in a single infection cycle: occlusion-derived virions (ODVs) and budded virions (BVs). ODVs are embedded in occlusion bodies (OBs) and are responsible for starting the primary infection in epithelial midgut cells and for host-to-host transmission, while BVs are responsible for spreading viruses throughout the body via the hemolymph [9,10]. Baculoviruses infect larval stages of insects, mainly of the order Lepidoptera. The baculovirus Spodoptera exigua multiple nucleopolyhedrovirus (SeMNPV) is highly infectious to its single host *Spodoptera exigua*, the beet armyworm, and is an important biocontrol agent of this species [11]. Apoptosis is observed in host insects infected by baculoviruses [2,12]. SeMNPV infection has been suggested to trigger apoptosis in the hemocytes of *S. exigua* larvae [12]. In addition, in the hemolymph and fat bodies of *S. exigua* larvae apoptosis was observed following infection by Spodoptera litura nucleopolyhedrovirus (SpltNPV) [12]. Baculoviruses contain pro-apoptotic genes, for example, the immediate early gene 1 (*ie1*) from the baculovirus Autographa californica multiple nucleopolyhedrovirus (AcMNPV). Previous studies have shown that the *ie1* gene from AcMNPV is involved in the formation of apoptotic bodies in virus-infected Sf21 cells by initiating virus DNA replication events that subsequently trigger cell death [7,13,14]. Apart from pro-apoptotic genes (like *ie1*), baculoviruses also contain anti-apoptotic genes. AcMNPV carries the anti-apoptotic gene *p35*, while other baculoviruses contain either *p35* homologues or inhibitor of apoptosis (*iap*) genes [15]. SeMNPV possesses *iap2* and *iap3* homologues [16]. SeMNPV IAP3 was able to block chemical induction of apoptosis in insect cells and mammalian cells; however, the function of *iap2* is not known yet [17,18].

SeMNPV, like bracoviruses, carries a phosphatase gene, in this case called protein tyrosine phosphatase 2 (*ptp2*). Both SeMNPV PTP2 and MdBV PTP-H2 contain a His–Cys (HC) signature motif in their catalytic site, which is characteristic for proteins belonging to the PTP superfamily. Moreover, the PTP2 protein carries a C-terminal consensus sequence that is characteristic for mitogen-activated protein kinase (MAPK) phosphatases. Several MAPK phosphatases have been reported to regulate MAPK pathways and are important regulators of apoptosis [19]. Here, we tested whether SeMNPV PTP2 has a similar function to the MdBV PTP-H2 in inducing apoptosis in its host. First, we investigated the apoptotic effect of PTP2 on cultured cells and on *S. exigua* larval hemocytes. Then we compared the budded virus titer, the virulence indices (including the viral infectivity and the time to death) and the OB yield between insects infected with either the wild type (WT) or the mutant virus lacking the *ptp2* gene. The results show that PTP2 functions as a pro-apoptotic protein in cultured cells and in larval hemocytes. Moreover, PTP2 contributes to a higher OB yield in larvae.

## 2. Materials and Methods

### 2.1. Insects, Cell Lines and Virus 

*Spodoptera exigua* larvae were reared on artificial diet as described before [20]. Sf21 cells (Sigma-Aldrich, Darmstadt, Germany) were maintained as monolayers in Grace’s medium (Invitrogen, Glasgow, UK) supplied with 10% fetal bovine serum (FBS) (Invitrogen) and 0.1% gentamycin (50 µg/mL, Invitrogen). Se301 cells, originally derived from *S. exigua* [21], were maintained as monolayers in CCM3 serum free medium (Hyclone, Cramlington, UK) supplied with 5% FBS and 0.1% gentamycin (50 µg/mL). The SeBac10 bacmid, derived from the SeMNPV US-1 strain [22], was used in this study.

### 2.2. Assessment of Apoptosis in Sf21 Cells

#### 2.2.1. Construction of Plasmids for Transient Expression Assays

The pIB-DEST expression vector (Invitrogen) was used for transient expression assays in Sf21 cells. The vector contains the constitutively expressed early OpIE2 promoter, derived from Orgyia pseudotsugata (Op) MNPV, to drive the expression of the gene of interest. Each gene to be expressed was cloned downstream of the *egfp* open reading frame (ORF), from which it was separated by the foot and mouth disease virus 2A ribosome skipping element (FMDV2A). EGFP was used to monitor the transfection efficiency. FMDV2A is a seventeen amino acids long (NFDLLKLAGDVESNPGP) element that allows co-translational cleavage between the marker protein (EGFP) and the protein to be analyzed. In total five pIB-DEST constructs were made: (i) modified pIB-DEST vector (pIB-DESTmod) in which the chloramphenicol resistance (*cat*) gene and the *ccdB* gene were replaced by a 888 bp long internal DNA segment from the AcMNPV *gp64* ORF (nt509-1396) to have a negative control vector that could be amplified in the *E. coli* strain DH5α (the *ccdB* gene in the original pIB-DEST is lethal to *E. coli*); (ii) pIB-EGFP encoding only EGFP; (iii) pIB-SePTP2 encoding EGFP and SeMNPV PTP2; (iv) pIB-SePTP2mut encoding EGFP and SeMNPV PTP2 containing a mutation in its catalytic site (mutation of cysteine to serine at position 110 (C110S)); and (v) pIB-AcPTP encoding EGFP and the AcMNPV PTP protein (distantly related to PTP2) [23], to allow comparison with a different viral protein tyrosine phosphatase. To construct these plasmids, the AcMNPV *gp64* segment and the *ptp* ORF were amplified from the AcMNPV E2 bacmid [24] using primer pairs 1 and 2, and 3 and 4, respectively (Table S1). The Se*ptp2* ORF was amplified from the SeMNPV bacmid SeBac10 using primers 5 and 6 (Table S1). To obtain the C110S catalytic mutant of Se*ptp2* a *ptp2^C110S^* mutation was created by a two-step PCR reaction using SeBac10 as template. First, primer 7 (Table S1), which introduced two point mutations at nucleotides 328 and 330 relative to the ATG start codon, was combined with primer 8 (Table S1), which annealed to the 3′ end of the *ptp2* ORF, to create a 3′ segment with the required mutations. The resulting 198 bp PCR product was used as reverse primer and combined with primer 9 (Table S1), annealing to the 5′ end of the *ptp2* ORF, to create the full length *ptp2^C110S^* insert, again with SeBac10 as a template. Then the *ptp2^C110S^* insert was verified by sequence analysis after cloning into the pJET1.2 cloning vector (Fermentas, Merelbeke, Belgium). The resulting plasmid pJET-*ptp2^C110S^* was then used as template for PCR using the primers 5 and 6 to allow Gateway cloning (see below).

The forward primers were designed to introduce an attB1 site and a *Hind*III site, while the reverse primers introduced an attB2 site (Table S1) to enable Gateway^®^ cloning (Invitrogen). All PCRs were performed with the proofreading Phusion polymerase (Finnzymes, Waltham, MA, USA). The resulting PCR amplicons were first cloned into the pDONR207 donor plasmid (Invitrogen) and sequenced and subsequently cloned into the pIB-DEST plasmid. The EGFP-FMDV2A was obtained as *Hind*III fragment from the plasmid CHIKrep-pac2AEGFP as described before [25] and inserted into *Hind*III-linearized pIB-DEST plasmids containing the genes of interest.

#### 2.2.2. Transient Expression Assay

In total, two transient expression assays were conducted and each assay was performed twice as two independent replicates. In the first assay the apoptotic effects of the above-mentioned pIB-DEST-derived plasmids on Sf21 cells was assessed. In total, seven treatments were included in this assay: (1) pIB-EGFP + Actinomycin D (ActD, Sigma-Aldrich), a chemical inducer of apoptosis, as a positive control; (2) pIB-SePTP2; (3) pIB-SePTP2mut; (4) pIB-AcPTP; (5) pIB-EGFP; (6) pIB-DESTmod, to monitor whether the pIB-DEST vector itself induces apoptosis on Sf21 cells; and (7) mock transfected cells (no vector used). The second transient expression assay was conducted to assess whether apoptosis inhibitors were able to inhibit Se*ptp2* induced apoptosis in Sf21 cells. In this case cells were transfected with pIB-SePTP2, in the presence or absence of the pan-caspase inhibitor carbobenzoxy-valyl-alanyl-aspartyl-(O-methyl)-fluoromethylketone (Z-VAD-FMK, Promega, Leiden, The Netherlands).

For both transfection assays Sf21 cells were seeded at a confluency of 25–30% in 6-well plates. The cells were incubated overnight and two h prior to transfection, the culture medium was replaced with Grace’s medium without serum. Transfections were performed with 4 µg of the pIB-DEST plasmids containing the genes of interest. Cellfectin II (Invitrogen) was used as the transfection reagent according to the manufacturers’ protocol. ActD was added to the medium 40 h post transfection (hpt) at a final concentration of 0.25 µg/mL. The caspase inhibitor Z-VAD-FMK was supplied in the normal medium at a concentration of 20 µM at 5 hpt. Cells were monitored daily using a Zeiss Axio Observer inverted fluorescence microscope to analyze EGFP expression and apoptotic body formation.

#### 2.2.3. Caspase Assay

A caspase assay (treatments) were performed using the experimental set-up for transient expression assay 1 described above; the assay was performed twice as two independent replicates. The transfection and apoptosis induction procedures were the same as described above, with the exception that all transfections were performed in 24-well plates, making each of the reagent volumes four times smaller. At 48 hpt cells were homogeneously resuspended in culture medium. For each sample, 50 µL of cell suspension was mixed with 50 µL of caspase-glo 3/7 substrate (Promega) in white 96-well plates. All samples were prepared *in duplo* per replicate. The plate was incubated in the dark for one hour at room temperature. Luminescence was measured in a fluorometer (Optima; settings: 5 flashes with maximal gain and top optic measurement). Luminescence was measured as relative luminescent units (RLUs). Differences in RLUs between the treatments were tested using the software package SPSS, version 22.0 (Armonk, NY, USA) [26]. Data were first tested for normality (Kolmogorov–Smirnov test) and homogeneity of group variance (Levene’s test). Where possible, logarithmic transformations were performed to attain normality and homogeneity of variances. A one-way analysis of variance (ANOVA) was performed to determine whether there were significant differences in RLUs among the different treatments. When a significant difference was found, pairwise comparisons were performed using Tukey *post hoc* tests.

### 2.3. Assessment of Apoptosis in *Spodoptera exigua* Hemolymph Cells

#### 2.3.1. Construction of Recombinant Virus

An SeBac10-derived bacmid with a partial deletion of the *ptp2* ORF (Δ*ptp2)* was constructed using the combined lambda RED and Cre-recombination methods described before [27]. The mutant had a deletion of the major part (354 bp) of the *ptp2* ORF (498 bp), ranging from nucleotide 48 to 401. Briefly, PCR products of the chloramphenicol (*cat*) resistance gene flanked by modified *loxP* sites and with 50 bp overhangs homologous to the flanking regions of the region to be deleted were generated with Phusion polymerase using primer 10 and 11 (Table S1). Subsequently, the major part of the *ptp2* ORF was replaced by the *cat* gene flanked by modified *loxP* sites. The *cat* gene was then removed by Cre-recombinase, leaving an inserted segment of 162 bp that contained the recombined *loxP* site. The deletion of the *cat* gene was checked by PCR using primers 12 and 13 (Table S1) that annealed to the 5′ and 3′ areas outside the deleted region. To enable oral infection of *S. exigua* larvae, the SeMNPV *polyhedrin* promoter and ORF were introduced into the Δ*ptp2* SeBac10, which, similar to the ancestral SeBac10, lacks the *polyhedrin* gene, as described before [20].

Recombinant Δ*ptp2* SeMNPV viral OBs were generated by injecting Δ*ptp2* SeBac10 bacmid DNA together with transfection reagent into 4th instar *S. exigua* larvae as described before [20]. Δ*ptp2* SeMNPV OBs were amplified in and purified from larvae using protocols as described before [20]. The concentration of OBs was determined using a Bürker–Türk hemocytometer (Marienfeld). SeBac10 derived WT virus (WT SeMNPV) [20] was used as a control.

#### 2.3.2. Apoptosis in *S. exigua* Larvae Hemocytes

Late 2nd instars of *S. exigua* were starved overnight for 16 h and allowed to molt. The next morning, newly molted 3rd instars were infected with WT SeMNPV or Δ*ptp2* SeMNPV using droplet feeding as described before [27]. Viral concentrations of 10^6^ OBs/mL, known to kill at least 90% of WT SeMNPV-infected larvae, were used for infection. A virus-free sucrose solution was used for mock infections. For each treatment, 48 larvae were infected and the experiment was performed twice as two independent replicates.

Apoptosis of hemocytes was measured by bleeding 3rd instar *S. exigua* larvae from a cut proleg 48 h post infection (hpi). Two microliter hemolymph was collected from each infected larva and the hemolymph samples from 10 larvae were pooled in 80 µL PBS containing 1-phenyl-2-thiourea (PTU, 0.1% final concentration) to prevent melanization. Next, 5 µL of Annexin V-EGFP (Annexin V-EGFP Apoptosis Detection Kit, BioVision, Milpitas, CA, USA) was added to stain any apoptotic cells, while Hoechst dye was added at a concentration of 10 µM to stain the nuclei of all cells. The mixtures were incubated at RT for 10 minutes and fluorescence was studied with a Zeiss Axio Observer inverted microscope.

#### 2.3.3. RNA Extraction and RT-PCR

To confirm the expression of *ptp2* in WT SeMNPV-infected larvae, and to confirm the absence of *ptp2* expression in ∆*ptp2* SeMNPV-infected larvae, an RT-PCR amplification was performed on total RNA extracted from infected single whole larvae at two days post infection (dpi). Infections of 3rd instar *S. exigua* larvae were performed using the same procedure and treatment as described above. At two dpi, a single larva was homogenized in 250 µL Trizol reagent (Invitrogen). Total RNA purification and subsequent cDNA synthesis were performed as described before [20]. RT-PCR was performed using primer pairs to amplify (i) a 486 bp sequence within the ORF of the *S. exigua* host translation initiation factor *eIF5A* [28] to verify that larval RNA was successfully extracted and that cDNA was synthesized (primer 14 and 15; Table S1); (ii) a 492 bp sequence within the coding sequence of the SeMNPV immediate early (*ie1*) gene to confirm successful virus infection (primer 16 and 17, Table S1); (iii) a 365 bp sequence encompassing the recombined *loxP* site to check for the correct deletion (the forward primer stretched from 27 bp upstream of the *ptp2* start codon to the first 9 bp of the *ptp2* ORF, the reverse primer annealed from 109 to 129 bp downstream of the *ptp2* stop codon) (primer 12 and 13; Table S1). For each sample, a non-RT control sample was included, in which water was added instead of reverse transcriptase in the RT step. In addition, for each PCR a negative control with only water and a positive control with purified WT SeBac10 as template were processed.

### 2.4. Comparison of Virus Infectivity and Ob Yield

#### 2.4.1. Virus Titrations

To confirm whether deletion of the *ptp2* gene affect BVs production in larval hemocytes, BVs titers in WT SeMNPV- and ∆*ptp2* SeMNPV-infected larvae were compared at two dpi. Infections were performed using the same procedures and treatments as described above. The experiment was performed three times as three independent replicates and per replicate 70 larvae were infected for each treatment. Two microliter of hemolymph was collected at 48 hpi from each larva as described above. Samples from 50 larvae were pooled in a total volume of 4 mL PBS containing 0.1% PTU and filtered through a 0.45 µm non-pyrogenic filter to remove hemocytes and potential microbial contaminants. The filtered sample was used for virus titration. The 50% tissue culture infectious dose (TCID_50_) was determined using an endpoint dilution assay (EPDA) on Se301 cells [29] and scored at 7 dpi for the presence of SeMNPV by looking at the cytopathic effect. The results were analyzed for significant differences in virus titers by a *t* test in GraphPad Prism 5 using a 95% confidence interval.

#### 2.4.2. Infectivity Assays

To see whether removal of the *ptp2* gene of SeMNPV affected viral infectivity, we performed infectivity assays to determine the infectivity of WT and Δ*ptp2* SeMNPV in 3rd instar *S. exigua* larvae as described before [20]. Five different concentrations were included for each virus, in 6-fold serial dilutions: 1.3 × 10^6^, 2.0 × 10^5^, 3.6 × 10^4^, 6.0 × 10^3^, and 1.0 × 10^3^ OBs/mL. Infections were performed as described above. Mock-infected larvae fed with a virus-free solution were used as controls. Larvae were scored for mortality from three dpi onwards until all larvae had died or pupated. Larvae that died for other reasons than virus infection were excluded from the analysis. The assays were performed three times. The program R v3.0.0 [30] was used for analyzing the data using a logistic regression model as described before [27]. Treatment was used as a fixed effect and the model followed a binomial distribution.

To compare the time to death for WT and Δ*ptp2* SeMNPV in 3rd instar *S. exigua* larvae, 48 larvae were infected with an concentration of 10^6^ OBs/mL, known to kill at least 90% of infected larvae as described above. Mock-infected larvae were included as controls. Larvae were checked for mortality twice per day as described above and three independent replicates were performed. The effects of treatment and experiment on time to death were analyzed using Cox’s proportional model in the program R as described before [27]. Since most larvae died as 3rd instars, larval stage was excluded as a factor in the model. LT_50_ values were calculated in R.

#### 2.4.3. Determination of OB Yield

Five larvae were randomly selected from each LT_50_ assay to determine the OB yield/larva. In total, 15 larvae were used for each virus (WT and Δ*ptp2* SeMNPV). The cadavers were individually homogenized in 0.5 mL sterile water and then filtered through a double layer of cheese cloth. The filtrate was centrifuged at 6000 rpm for 5 min. The supernatant was discarded and the pellet was resuspended in 0.5 mL sterile water. OB yield/larva was calculated by counting the number of OBs in 10 µL virus solutions using a Bürker–Türk hemocytometer. The counting was performed three times and the average was used for calculating the OB yield per larva. The OB yield was analyzed (virus treatment was used as a fixed factor) by a *t* test in GraphPad Prism 5 using a 95% confidence interval.

## 3. Results

### 3.1. Transient Expression of SeMNPV Ptp2 in Sf21 Cells Induces Mild Apoptosis

**Figure 1:**
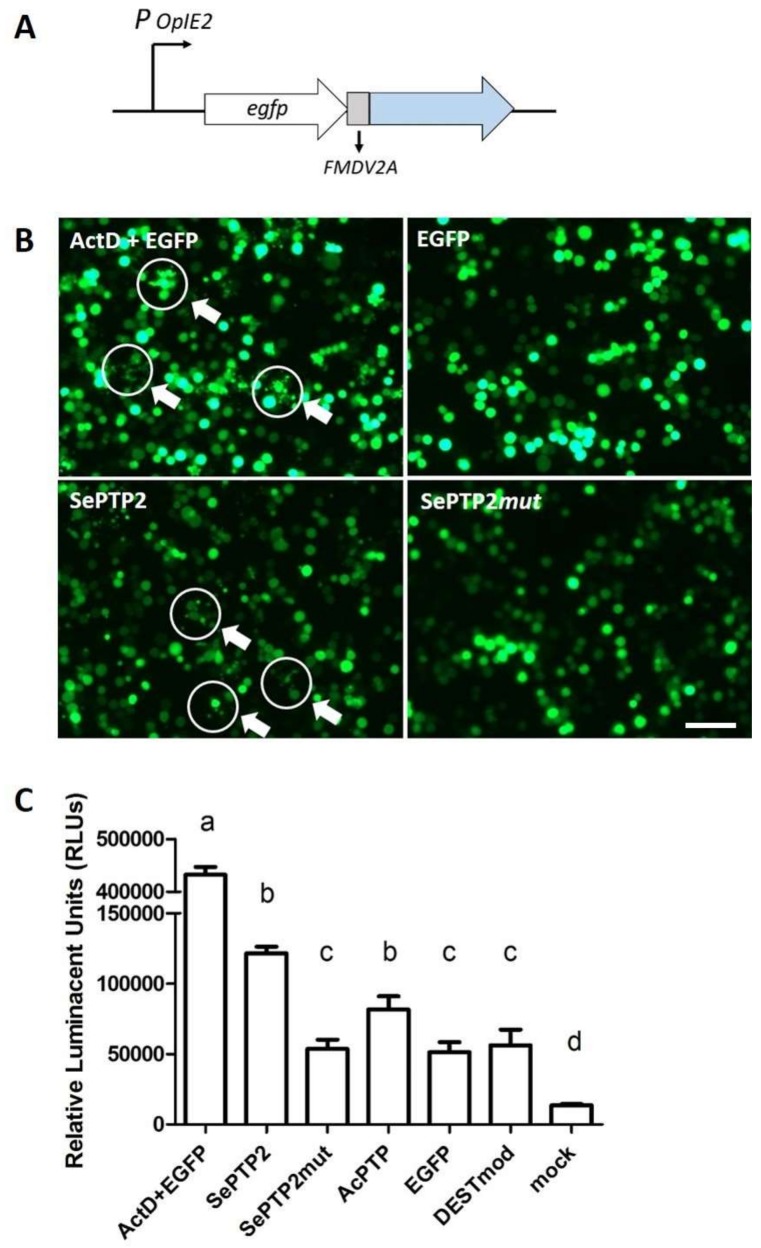
Transient expression of the *Spodoptera exigua* protein tyrosine phosphatase 2 (SePTP2) induced mild apoptosis in Sf21 cells. (**A**) Overview of the expression cassette in the pIB-DEST vector: *egfp* and the gene of interest were separated by the foot and mouth disease virus (FMDV) 2A region and expression of the cassette was driven by the OpIE2 promoter; (**B**) EGFP expression in Sf21 cells at 48 h post-transfection (hpt) with pIB-EGFP + ActD (ActD + EGFP), pIB-EGFP (EGFP), pIB-SePTP2 (SePTP2), or pIB-SePTP2mut (SePTP2mut), respectively. Apoptotic bodies are indicated by white circles and arrows, scale bar = 200 µm; (**C**) caspase activity levels in Sf21 cells transfected with pIB-EGFP + ActD (ActD + EGFP), pIB-SePTP2 (SePTP2), pIB-SePTP2mut (SePTP2mut), pIB-AcPTP (AcPTP), pIB-EGFP (EGFP), pIB-DESTmod (DESTmod) and mock. Caspase 3/7 activity in transfected Sf21 cells was measured as relative luminescent units (RLUs) at 48 hpt, relative to a blank containing cell medium. Error bars represent the standard error of the mean. Treatment groups marked with a different letter (a, b, c or d) are significantly different (Table S2).

Since the protein tyrosine phosphatase gene (*ptp-H2*) from the polydnavirus MdBV showed pro-apoptotic effects on host cells, we hypothesized that SeMNPV *ptp2* might function in a similar way. To investigate the pro-apoptotic effects of the SeMNPV *ptp2* gene, we performed transient expression assays in Sf21 cells, using plasmids from which *ptp2* was expressed together with EGFP (see Figure 1A). Based on the number of EGFP-expressing cells, transfection efficiencies were estimated to be 40—50% for each tested plasmid. The negative controls included mock-transfected cells, cells transfected with the modified ‘empty’ expression vector pIB-DESTmod (to check whether expression vector itself induces apoptosis in transfected cells), and cells transfected with pIB-EGFP expressing only EGFP, and in these treatments the cells did not show signs of apoptosis (Figure 1B; Figure S1). As a positive control, cells transfected with pIB-EGFP were treated with the strong apoptosis inducer ActD and morphological changes characteristic for apoptotic cells (cell blebbing and formation of apoptotic bodies) were observed (Figure 1B; Figure S1). Similarly, cell blebbing and apoptotic bodies were observed in cells transfected with pIB-SePTP2, but to a lesser extent compared with cells treated with ActD (Figure 1B; Figure S1). In contrast, cells transfected with pIB-SePTP2mut, expressing a catalytic mutant of PTP2, did not show the formation of apoptosis and appeared healthy (Figure 1B; Figure S1). Signs of apoptosis were not observed in cells transfected with pIB-AcPTP, coding for the distantly related AcMNPV PTP gene (Figure S1). These results indicate that transient expression of SeMNPV PTP2 caused apoptosis in Sf21 cells. Since transfection with the catalytic mutant did not result in apoptotic cells, we conclude that the phosphatase activity of SePTP2 is needed to induce apoptosis.

### 3.2. SePTP2 Induces Apoptosis by Caspase Activation

Caspases are a family of cysteine proteases that play an essential role during the induction of apoptosis. To study whether caspases are activated in the presence of PTP2, we measured the activity of the effector caspases 3 and 7 at 48 hpt. This activity was quantified by interaction of the effector caspases 3 and 7 with caspase-glo 3/7 substrates, which generates a luminescent signal (expressed in RLUs). The positive control expressing EGFP in the presence of ActD (ActD + EGFP) showed the highest caspase activity, corresponding to approximately 430,000 RLUs (Figure 1C). This level of activity was significantly higher compared to all other treatments (*P* < 0.001 for all comparisons; Table S2). Cells expressing SePTP2 showed caspase activity levels of approximately 120,000 RLUs (Figure 1C), which was significantly lower than for the ActD + EGFP treatment (*P* < 0.001), but significantly higher than for the other treatments (*P* < 0.005 for all comparisons; Table S2), except for AcPTP (*P* = 0.149). Mutation of the SePTP2 catalytic domain decreased the caspase activity level significantly to approximately 54,000 RLUs (*P* < 0.001). Cells transfected with pIB-EGFP and pIB-DESTmod, showed similar low levels of caspase activity as cells transfected with pIB-SePTP2mut (Figure 1C; *P* = 1.000 for both comparisons). For both replicates, cells transfected with a pIB-DEST vector (ActD + EGFP, SePTP2, SePTP2mut, AcPTP, EGFP, or DESTmod) showed higher levels of caspase activity than the mock transfection (Figure 1C; *P* < 0.001 for all comparisons; Table S2). The results indicate that caspase activities were significantly higher in cells transfected with pIB-SePTP2 than cells transfected with other plasmids (except for cells transfected with pIB-AcPTP), supporting the conclusion that SePTP2, but also AcPTP, induces mild apoptosis upon transient expression in Sf21 cells. However, for AcPTP, no visual signs of apoptosis (apoptotic bodies or cell blebbing) were observed (Figure S1).

**Figure 2:**
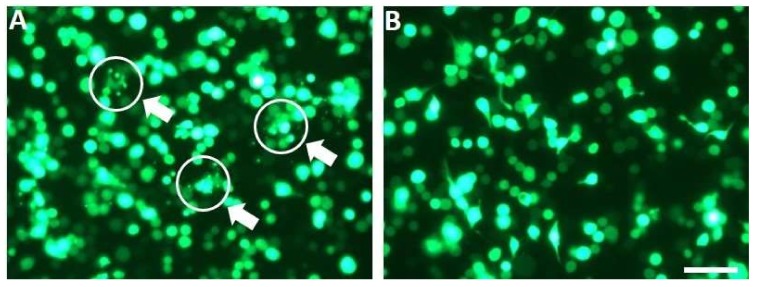
Caspase inhibitor carbobenzoxy-valyl-alanyl-aspartyl-(O-methyl)-fluoromethylketone (Z-VAD-FMK) blocked induction of apoptosis in Sf21 cells expressing SePTP2. EGFP expression in Sf21 cells at 48 h post transfection with pIB-SePTP2 (**A**) or with pIB-SePTP2 + Z-VAD-FMK (**B**). Apoptotic bodies are indicated by white circles and arrows, scale bar = 200 µm.

To confirm the involvement of caspases in SePTP2-induced apoptosis, we repeated the transfection experiment with pIB-SePTP2 in the presence or absence of the caspase inhibitor Z-VAD-FMK. In the absence of caspase inhibitor, cells transfected with pIB-SePTP2 again showed induction of apoptosis (Figure 2A). However, the presence of the caspase inhibitor blocked the induction of apoptosis: cells appeared to be healthy and cell blebbing and apoptotic bodies were not observed (Figure 2B). These data provide further evidence that SePTP2 induces apoptosis in Sf21 cells via caspase activation.

### 3.3. Deletion of the Ptp2 Gene Reduces SeMNPV-Induced Apoptosis in S. exigua Hemocytes

**Figure 3:**
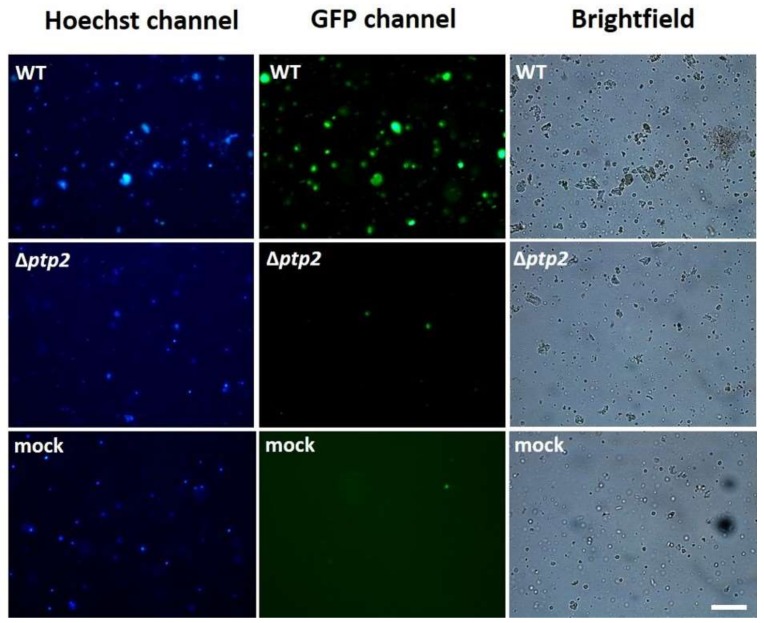
Fluorescence microscopy analysis of cells in *S. exigua* hemocytes at 48 h post transfection. Cells were stained with Hoechst (left panel) and Annexin V-EGFP (middle panel). Cells were obtained from larvae infected with wild-type (WT) Spodoptera exigua multiple nucleopolyhedrovirus (SeMNPV), ∆*ptp2* SeMNPV or no virus (mock), scale bar = 200 µm.

To investigate whether SePTP2 also has a pro-apoptotic effect in hemocytes of *S. exigua* larvae, a mutant SeMNPV virus lacking the *ptp2* gene (∆*ptp*2 SeMNPV) was created. Hemocytes were collected from WT and ∆*ptp*2 SeMNPV-infected larvae at 48 hpi and then stained with Annexin V-EGFP (to stain apoptotic cells) and Hoechst (to stain the nuclei of all collected cells). Fluorescence microscopy analysis showed that WT SeMNPV also induced apoptosis in *S. exigua* hemocytes: The green Annexin V-EGFP signal was observed in many cells, indicating that these cells went into apoptosis (Figure 3). In contrast, hemocytes isolated from ∆*ptp*2 SeMNPV-infected and mock-infected larvae showed a much lower number of green cells, indicating apoptosis was induced at a much lower level by these treatments (Figure 3).

**Figure 4:**
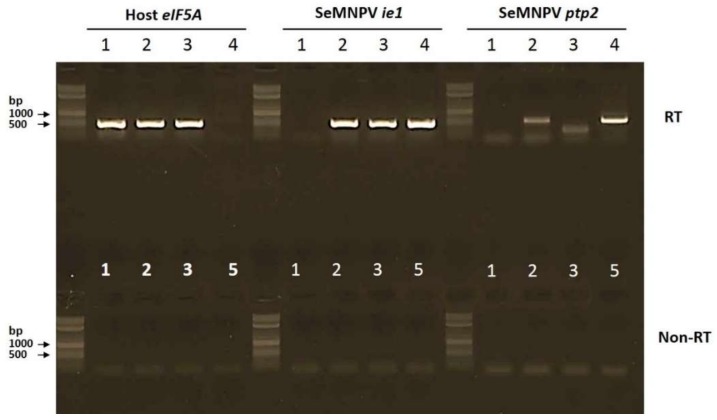
The full-length *ptp2* gene is expressed in WT SeMNPV-infected but not in ∆*ptp2* SeMNPV-infected *S. exigua* larvae. RT-PCR analysis of mock-infected (1), WT SeMNPV-infected (2), or ∆*ptp2* SeMNPV-infected (3), *S. exigua* larvae processed for RT-PCR analysis at two days post infection. For each PCR a WT SeMNPV bacmid control (4) and a water control (5) were included. Expression of the host *eIF5A* gene, the SeMNPV *ie1* gene and the SeMNPV *ptp2* gene were analyzed. For each RT sample, a PCR without RT step (non-RT) was performed in parallel. The 2-Log DNA Ladder (0.1–10.0 kb, New England BioLabs Inc. Ipswich, MA, USA) was used in the agarose gel to estimate PCR fragment sizes.

To confirm the expression of *ptp2* in WT SeMNPV-infected larvae and to verify the absence of a full-length *ptp2* transcript in ∆*ptp2* SeMNPV-infected larvae, an RT-PCR was performed on total RNA extracted from mock- and virus-infected individual larvae at two dpi. As expected, a full-length *ptp2* transcript was present in WT SeMNPV-infected larvae and absent in mock-infected larvae (Figure 4, right panel, lanes 1 and 2). Partial deletion of 354 bp within the *ptp2* ORF in ∆*ptp2* SeMNPV resulted in a smaller product (Figure 4, right panel, lane 3) The SeMNPV *ie1* gene, included as a control to check for successful virus infection (Figure 4, middle panel), was expressed in all virus-infected larvae and absent in mock-infected larvae. An RT-PCR targeting the mRNA of the *S. exigua eIF5A* gene was included as a control for correct RNA extraction and cDNA synthesis, and as expected *eIF5A* was expressed in both virus- and mock-infected larvae (Figure 4, left panel).

### 3.4. Virus Infectivity Was Not Affected by Deleting the Ptp2 Gene

To determine whether deletion of the *ptp2* gene affected the production of infectious budded viruses (BVs), hemolymph of WT and ∆*ptp2* SeMNPV-infected larvae was collected at 48 hpi and the infectious BV titer was measured as the TCID_50_ value for Se301 cells for three biological replicates. The BV titers were not significantly different between the two treatments at 48 hpi (*t* test = 1.314; d.f. = 4; *P* = 0.2592), indicating that deletion of the *ptp2* gene did not affect the production of infectious BVs (Table S3).

To study whether deletion of the viral *ptp2* gene affected viral infectivity, we performed a logistic regression on the mortality data obtained after infecting *S. exigua* third instar larvae with WT and ∆*ptp2* SeMNPV, for each replicate separately. The ratio of infectivity of ∆*ptp2* SeMNPV to WT SeMNPV (odds ratio) was determined. In all three replicates, the infectivity of WT and ∆*ptp2* SeMNPV were not significantly different (judged by overlap of 95% confidence interval of the odds ratio; Table S4). There were no significant differences in mortality between the three replicates.

We then investigated whether deletion of the viral *ptp2* gene affected the time to death for infected third instars of *S. exigua*, using a survival analysis. The Cox’s proportional hazards model was used to determine the mortality rate (hazard rate, rate at which larvae died) for WT and ∆*ptp2* SeMNPV. Treatment and experiment were included as factors. The mortality rate for larvae infected with mutant virus was similar to that of WT SeMNPV-infected larvae (ratio of 1.05; *z* = 0.375; *P =* 0.721). There were no significant differences between the three replicates.

### 3.5. Deletion of the Ptp2 Gene Decreases SeMNPV OB Yield

**Figure 5:**
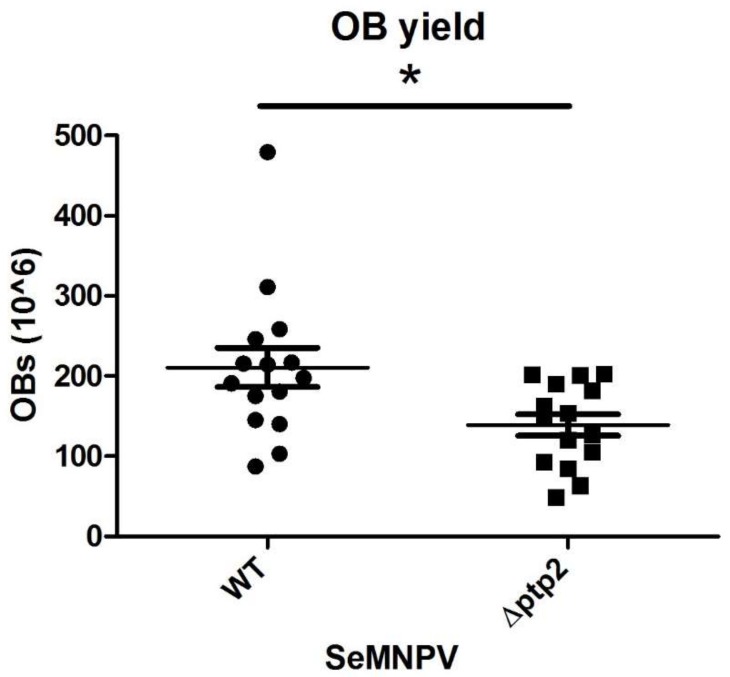
Occlusion body (OB) yield is significantly reduced in *S. exigua* larvae infected with ∆*ptp2* SeMNPV compared to larvae infected with WT SeMNPV. Data points represent the OB yield of individual larvae. Horizontal lines show the mean value of OB yield and whiskers the standard error of the mean. Asterisk indicates significant difference (*t* test, *P* < 0.05).

To determine the effect of deletion of the *ptp2* gene on the total OB yield, we randomly selected 15 cadavers from WT and ∆*ptp2* SeMNPV-deceased larvae. The OB yield of individual larvae was counted using a Bürker–Türk hemocytometer. The OB yield from larvae killed by WT SeMNPV was significantly higher that from larvae killed by ∆*ptp2* SeMNPV, in which the OB yield was decreased by 34% (Figure 5; *t* test = 2.583; *d.f.* = 28; *P* < 0.05). Though deletion of the *ptp2* gene did not affect virus infectivity nor the time to death, the OB yield was lower for larvae infected with the mutant SeMNPV lacking *ptp2*.

## 4. Discussion

Many viruses carry genes that are involved in inducing apoptosis in host-derived cell lines [4,5,13,15,31], such as the *ptp-h2* gene from MdBV, which induces apoptosis in Sf21 cells [5]. The baculovirus SeMNPV carries a protein tyrosine phosphatase gene (*ptp2*) which contains the same catalytic domain as *ptp-h2*, therefore, we tested whether SeMNPV *ptp2* has a pro-apoptotic function as well. To test this hypothesis we took advantage of Sf21 cells, which are known to undergo apoptosis upon different stimuli [32]. Sf21 cells transfected with a plasmid expressing SePTP2 showed morphological signs of apoptosis and increased levels of caspase activity. However, the pro-apoptotic effect of SePTP2 might be restricted to certain cell types. Similar results were found for MdBV PTP-H2 as well; PTP-H2 induced apoptosis in Sf21 cells, but not in granulocytes or plasmatocytes of *S. frugiperda* larvae, *T. ni* High Five cells or *Drosophila* S2 cells [5]. More interestingly, some viruses even induce apoptosis in one cell type, but block apoptosis formation in another cell type. For example, Herpes simplex virus (HSV) induces apoptosis in Jurkat cells, a T-cell leukemia line, but protected HEp-2 cells (a carcinoma cell line) from apoptosis triggered by tumor necrosis factor alpha [31]. To our knowledge, there is no explanation so far why a viral gene induces apoptosis in one cell type, but not in another cell type. A possible explanation is that certain regulatory pathways are not active in cell types in which apoptosis is not induced. As a result, the pro-apoptotic effect of viral genes is restricted to certain cell types or host tissues.

Our data showed a significantly increased caspase activity in transfected cells expressing SePTP2. In addition, the phosphatase activity of SePTP2 was needed to activate the effector caspases. Cells expressing the distantly related baculovirus PTP (AcPTP) also showed increased caspase activity; however, apoptosis was not observed in these cells (absence of cell blebbing and apoptotic bodies), indicating that the pro-apoptotic effect was not an overall effect of transient expression of baculovirus PTPs. Similar results were also observed for MdBV; MdBV encodes 13 different PTPs, but only PTP-H2 showed a pro-apoptotic effect in Sf21 cells [5,33]. SePTP2 protein possesses a C-terminal consensus sequence that is characteristic for MAPK phosphatases, which remove the phospho-residue from MAPKs. There are three major MAPK pathways: extracellular signal-regulated kinase (ERK), c-Jun N-terminal kinase (JNK) and p38. All three pathways have been reported to be involved in regulating apoptosis [34,35,36]. Therefore, SePTP2 may induce apoptosis by regulating MAPK activity in cells. Further studies are needed to identify the substrates of SePTP2 to confirm this.

In our study, deletion of the *ptp2* gene severely reduced SeMNPV-induced apoptosis in *S. exigua* hemocytes, while the SeMNPV BV titer, virus infectivity and time to death were not affected upon deletion of *ptp2*. However, the OB yield was significantly higher from larvae infected with WT SeMNPV than from larvae infected with ∆*ptp2* SeMNPV. A previous study on BmNPV showed that ERK- and JNK-dependent signaling pathways contribute to BmNPV virus yield; knocking down the *Bombyx mori erk* or *jnk* genes reduced the production of both OBs and BVs [37]. However, whether (and which) viral genes are involved in activating the host ERK- and JNK-pathways during a BmNPV infection is not known. SePTP2 may function as a MAPK phosphatase that regulates host MAPK pathways that eventually affect OB yield in infected hosts. Virus-induced apoptosis is normally regarded as a defense mechanism against virus infection; however, some viruses stimulate apoptosis in immune cells to maximize viral fitness and transmission [38,39]. For example, the influenza A virus (IAV) induces apoptosis in innate immune cells at the early stage of infection to subvert host immunity [39]. Similar results were also found in MdBV infected *S. frugiperda* larvae [5]. We hypothesize that the induction of apoptosis by SeMNPV PTP2 suppresses the *S. exigua* larval immune system and by doing so the infected larvae accumulate more OBs. These higher OB yields most likely benefit virus dissemination, and hence transmission.

It seems contradictory that viruses carry both anti-apoptotic and pro-apoptotic genes. Baculoviruses are known to produce anti-apoptotic proteins (e.g., P35 and IAP) to suppress or delay apoptosis [15]. For example, besides SePTP2, SeMNPV also encodes the anti-apoptotic proteins IAP2 and IAP3 [17,18]. However, anti-apoptotic and pro-apoptotic genes may function via different pathways. While anti-apoptotic genes counteract host-induced apoptosis (which interferes with viral replication in general), allowing the virus to replicate and eventually increasing virus progeny, pro-apoptotic genes might serve to suppress the host immune system by targeting specific cell types. Future studies should show whether the induction of apoptosis in *S. exigua* larvae is restricted to hemocytes.

Overall, we conclude that the SeMNPV *ptp2* gene functions as a pro-apoptotic gene in cultured Sf21 insect cells, inducing mild apoptosis, and that the phosphatase activity of SePTP2 is needed for this process. Furthermore, we showed that the SeMNPV *ptp2* gene is involved in inducing apoptosis in host hemocytes. Viral suppression of host immunity eventually contributes to a higher OB yield.

## Supplementary Materials

The following are available online at http://www.mdpi.com/1999-4915/10/4/181/s1, Figure S1: Transient expression of SePTP2 induced apoptosis in Sf21 cells, Table S1: Primers used in this study, Table S2: P values of the one-way ANOVA, Table S3: TCID50 values of BVs, Table S4: Outcome of the logistic regression analysis of the infectivity assays.
