# Supplementary material for: Baculovirus PTP2 Functions as a Pro-Apoptotic Protein
Source: Viruses. 2018 Apr 7;10(4):181. doi: 10.3390/v10040181 (PMC5923475; doi:10.3390/v10040181)
Supplement: Supplementary file 1 [file viruses-10-00181-s001.zip › Figure S1.pdf]

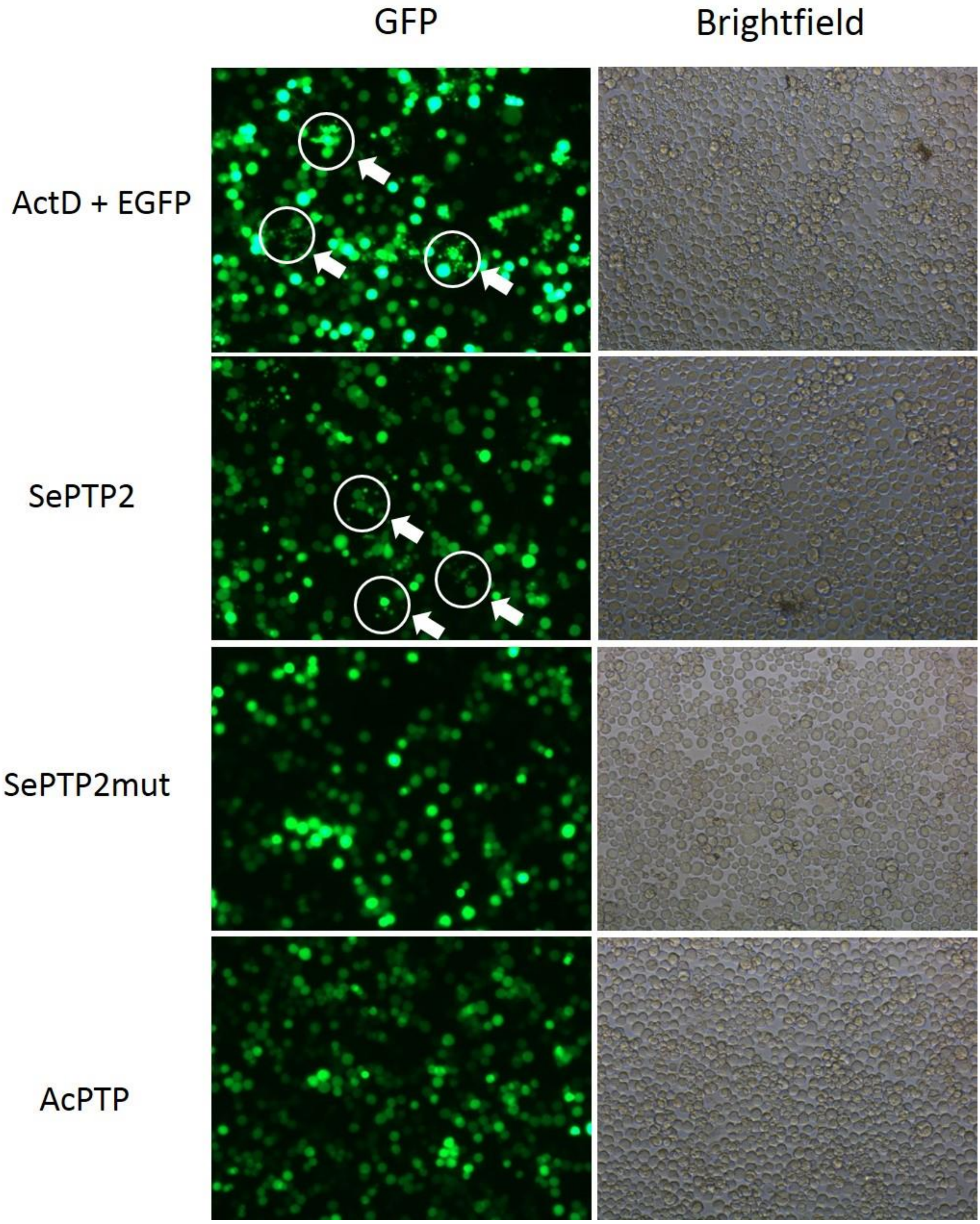
GFP
Brightfield
ActD + EGFP
SePTP2
SePTP2mut
AcPTP

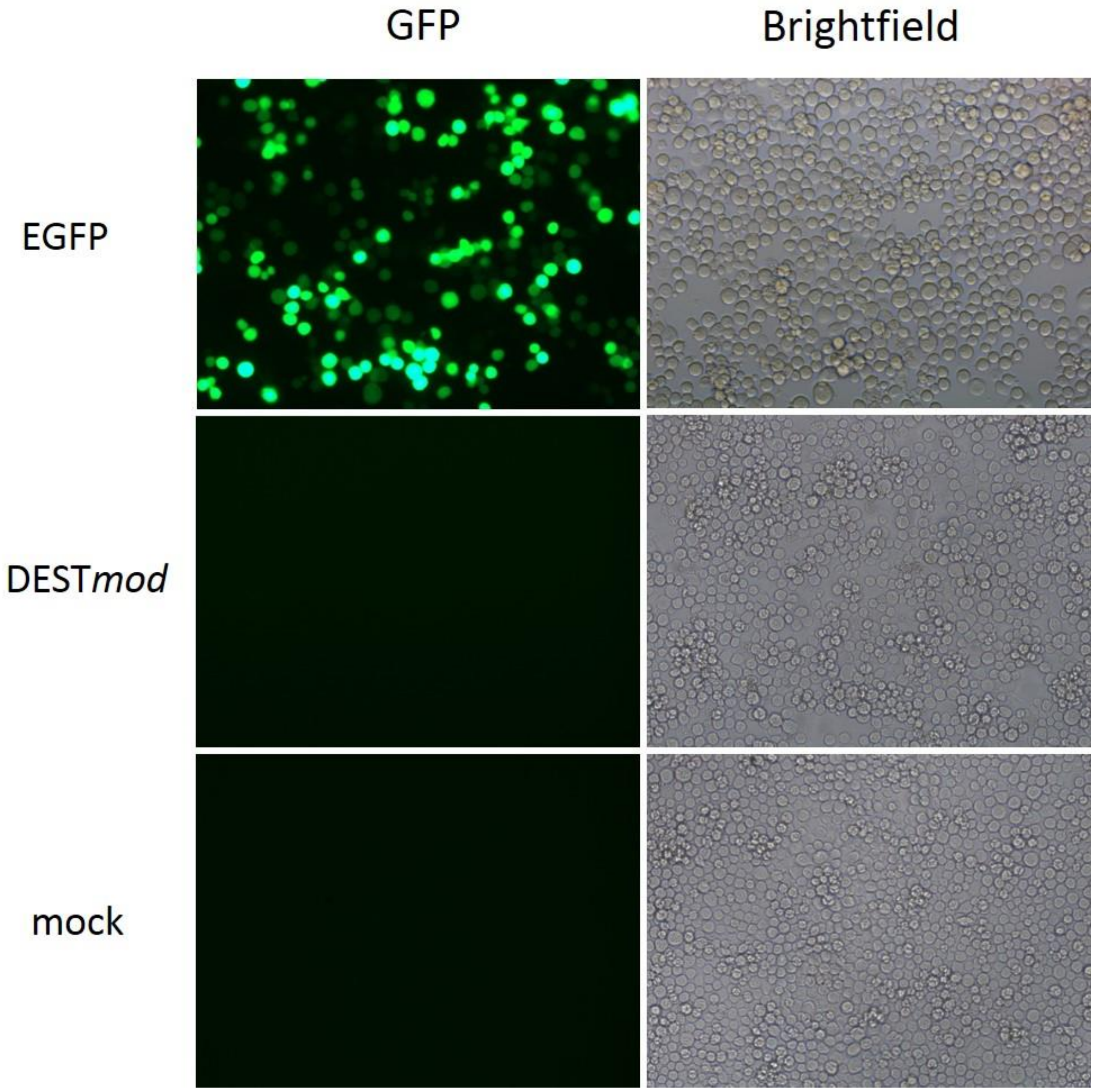

Figure S1. Transient expression of SePTP2 induced apoptosis in Sf21 cells. EGFP expression in Sf21 cells at 48 hours post transfection with pIB-EGFP + ActD (ActD + EGFP), pIB-SePTP2 (SePTP2), pIB-SePTP2mut (SePTP2mut), pIB-AcPTP (AcPTP), pIB-EGFP (EGFP), pIB-DESTmod (DESTmod) and mock. Apoptotic bodies are indicated by white circles and arrows.
