# Supplementary material for: Baculovirus PTP2 Functions as a Pro-Apoptotic Protein
Source: Viruses. 2018 Apr 7;10(4):181. doi: 10.3390/v10040181 (PMC5923475; doi:10.3390/v10040181)
Supplement: Supplementary file 1 [file viruses-10-00181-s001.zip › Table S1.pdf]

Table S1. Primers used in this study.

Primer number	Primer name	Sequence	Aim
1	pIB-DEST*mod*-fw	GGGG***ACAAGTTTGTACAAAAAAGCAGGCTGGG***AAGCTTAAATGTACAGCAGGCTC	Amplify Ac*gp64* segment with attB1 and HindIII site
2	pIB-DEST*mod*-rv	GGGG***ACCACTTTGTACAAGAAAGCTGGGT***CATTCAGACTGGTGCCGACG	Amplify Ac*gp64* segment with attB2 site
3	pIB-Ac*ptp*-fw	GGGG***ACAAGTTTGTACAAAAAAGCAGGCTGGG***AAGCTTATGTTTCCCGCGCGT	Amplify Ac*ptp* ORF with attB1 and HindIII site
4	pIB-Ac*ptp*-rv	GGGG**ACCACTTTGTACAAGAAAGCTGGGT**TTAAATTAATAAATCTTGAACGTAATTTTGTC	Amplify Ac*ptp* ORF with attB2 site
5	pIB-Se*ptp2*-fw	GGGG***ACAAGTTTGTACAAAAAAGCAGGCTGGG***AAGCTTATGCATACTAACGACGACAAC	Amplify Se*ptp2 and* Se$ptp2^{C110S}$ ORF with attB1 and HindIII site
6	pIB-Se*ptp2*-rv	GGGG***ACCACTTTGTACAAGAAAGCTGGGT***TCAAAATTCCATTTGTGATTCGGC	Amplify Se*ptp2 and* Se$ptp2^{C110S}$ ORF with attB2 site
7	$ptp2^{C110S}$-fw1	GGCGAGGGCAAAAGGGTGTACGTTCAT**A**G**C**CATGCGGGCGTGTCG	Create C110S mutation in SePTP2 catalytic site
8	$ptp2^{C110S}$-rv	TCAAAATTCCATTTGTGATTC	Amplify Se*ptp2* ORF
9	$ptp2^{C110S}$-fw2	ATGCATACTAACGACGACAAC	Amplify Se*ptp2* ORF
10	*Δptp2*-fw	ATTATAGTTTACGTCGCCGACGAAACAATCATTGATATCGTGGACACGAGCTCGGATCCACTAGTAACGG	Create *ptp2* ORF deletion
11	*Δptp2*-rv	ACTATGCATACTAACGACGACAACTTTACGCAACTGTCCAACGGAAACTGCCTCTAGATGCATGCTCGAG	Create *ptp2* ORF deletion
12	*Δptp2*-check-fw	ATGTGTGCTCTTCGTCAGATG	Check *ptp2* ORF deletion and RT-PCR on SeMNPV *ptp2*
13	*Δptp2*-check-rv	TTCAATATAATAAGAAGAACACTATGCATACT	Check *ptp2* ORF deletion and RT-PCR on SeMNPV *ptp2*
14	*Se-eIF5A*-fw	GCCATGGCTGACATCGAGGATAC	RT-PCR on *S. exigua eIF5A*
15	*Se-eIF5A*-rv	GCGGTACCGGTTTATTTGTCGAGAGC	RT-PCR on *S. exigua eIF5A*
16	*Se-ie1*-fw	GACAAGAATGACGATGATATCGG	RT-PCR on SeMNPV *ie1*
17	*Se-ie1*-rv	GGACAATTGCTTTTCCGAAAAC	RT-PCR on SeMNPV *ie1*
