# Supplementary material for: Baculovirus PTP2 Functions as a Pro-Apoptotic Protein
Source: Viruses. 2018 Apr 7;10(4):181. doi: 10.3390/v10040181 (PMC5923475; doi:10.3390/v10040181)
Supplement: Supplementary file 1 [file viruses-10-00181-s001.zip › Table S2.pdf]

Table S2. *P* values of the one-way ANOVA of the relative luminescent units in the caspase assays. Values are given for each comparison.

	ActD+EGFP	SePTP2	AcPTP	SePTP2mut	EGFP	DESTmod	mock
ActD+EGFP	---						
SePTP2	$P < 0.001$	---					
AcPTP	$P < 0.001$	$P = 0.149$	---				
SePTP2mut	$P < 0.001$	$P < 0.001$	$P = 0.139$	---			
EGFP	$P < 0.001$	$P < 0.001$	$P = 0.079$	$P = 1.000$	---		
DESTmod	$P < 0.001$	$P < 0.005$	$P = 0.176$	$P = 1.000$	$P = 1.000$	---	
mock	$P < 0.001$	$P < 0.001$	$P < 0.001$	$P < 0.001$	$P < 0.001$	$P < 0.001$	---
