# Supplementary material for: Baculovirus PTP2 Functions as a Pro-Apoptotic Protein
Source: Viruses. 2018 Apr 7;10(4):181. doi: 10.3390/v10040181 (PMC5923475; doi:10.3390/v10040181)
Supplement: Supplementary file 1 [file viruses-10-00181-s001.zip › Table S3.pdf]

Table S3. $TCID_{50}$ values of BVs isolated from the hemolymph of WT and Δ*ptp2* SeMNPV-infected larvae at 48 hpi.

	Replicate 1 ($TCID_{50}$/ml)	Replicate 2 ($TCID_{50}$/ml)	Replicate 3 ($TCID_{50}$/ml)
WT SeMNPV	$8.91*10^6$	$1.20*10^7$	$2.11*10^7$
Δ*ptp2* SeMNPV	$1.99*10^7$	$1.58*10^7$	$4.47*10^7$
