# Supplementary material for: Baculovirus PTP2 Functions as a Pro-Apoptotic Protein
Source: Viruses. 2018 Apr 7;10(4):181. doi: 10.3390/v10040181 (PMC5923475; doi:10.3390/v10040181)
Supplement: Supplementary file 1 [file viruses-10-00181-s001.zip › Table S4.pdf]

Table S4. Outcome of the logistic regression analysis of the infectivity assays. Per replicate, an odds ratio (relative potency) was determined: the ratio of the infectivity of Δ*ptp2* SeMNPV relative to the infectivity of WT SeMNPV. The upper and lower limits of the 95% confidence interval are also given, as well as the *P* value.

**Replicate**	**Viruses**	**Odds ratio**	**95% Confidence Interval**		***P* value**
			Low	High	
1	WT SeMNPV	1.000	-	-	-
	Δ*ptp2* SeMNPV	1.616	0.946	1.419	0.081
2	WT SeMNPV	1.000	-	-	-
	Δ*ptp2* SeMNPV	1.393	0.876	2.225	0.163
3	WT SeMNPV	1.000	-	-	-
	Δ*ptp2* SeMNPV	1.120	0.687	1.831	0.649
